# Flexible ligated ruthenium(II) self-assemblies sensitizes glioma tumor initiating cells *in vitro*

**DOI:** 10.18632/oncotarget.19028

**Published:** 2017-07-05

**Authors:** Palani Elumalai, Neha Kaushik, Dong Hwan Kim, Hyunuk Kim, Su Jae Lee, Eun Ha Choi, Ki-Whan Chi, Nagendra Kumar Kaushik

**Affiliations:** ^1^ Department of Chemistry, University of Ulsan, Ulsan, Republic of Korea; ^2^ Plasma Bioscience Research Center, Department of Electrical and Biological Physics, Kwangwoon University, Seoul, Republic of Korea; ^3^ Department of Life Science, Hanyang University, Seoul, Republic of Korea; ^4^ Energy Materials and Convergence Research Department, Korea Institute of Energy Research, Daejeon, Republic of Korea; ^5^ Department of Chemistry, Texas A&M University at Qatar, Doha, Qatar

**Keywords:** self-assembly, flexible ligand, arene-ruthenium, metallacycle, solid cancer

## Abstract

The tumorigenic potentials of residual cancer stem-like cells within tumors represent limitations of current cancer therapies. Here, the authors describe the effects of synthesized flexible, ligated, supramolecular self-assembled chair type tetranuclear ruthenium (II) metallacycles (2–5) on glioblastoma and glioma stem like cells. These self-assemblies were observed to be selectively toxic to glioma cells and CD133-positive glioma stem like cells population. Of the self-assembled compounds tested, metallacycle 4 more efficiently induced glioma stem like cells death within a brain cancer cell population and simultaneously inhibited the formation of free-floating gliospheres by reducing the sphere size. Detailed cell death studies revealed that treatment with metallacycle 4 reduced mitochondrial membrane potentials (an indicator of apoptosis) of glioma stem like cells. These results shows the elimination of cancer stem-like cells using an appropriate ligand binding adaptor offers a potential means of developing metal-based compounds for the treatment of chemo-resistant tumors.

## INTRODUCTION

The construction of well-defined supramolecular self-assembly architectures provides a strategy for the development of more efficient and robust materials for use in energy storage devices and biomedicine [1–6]. Indeed, a number of self-assembly strategies have been used to synthesize metalla-supramolecular structures for use in various devices as sensors, functional smart-materials, and molecular catalysts [7, 8]. Such, self-assembly processes are determined by the several factors, such as, types of organic ligands, metal precursors, counter anions, and solvents and the processing conditions used. External guest molecules, solvent polarity and concentration control In the formation of assemblies [9–12]. However, the influences of these factors on assembly and their modes of action have not been fully determined. Self-assemblies of rigid bis-pyridyldiimide derivatives have shown potential as antitumor agents and to bind to DNA and proteins [13]. The use of flexible donor components is less common in self-assembly process and only a few examples that employ a flexible organic spacer to create discrete building blocks have been described, and although flexible diimide based supramolecular self-assemblies have been produced, application studies are limited [14].

For more than a decade, one of us and others have explored metalla supramolecular self-assembly of half-sandwich building blocks incorporating arene-ruthenium complexes in terms of their syntheses, structural aspects, and biomedical applications [15–20]. These ruthenium-based complexes have attracted the attentions of those working in the cancer treatment research field. Ruthenium-based complexes are considered to be of chemotherapeutic importance because they have few side effects and are not prone to the development of drug resistance [21–25] In fact, some ruthenium-based complexes are undergoing clinical trials [26, 27] or at the preclinical development stage [28] due to their unique binding affinities for biomolecules [29].

Biologically active ruthenium-based small molecules have recently prompted studies of ruthenium supramolecular coordination complexes with a particular focus on their antitumor efficacies. As a result, a number of ruthenium complexes with anticancer properties have been self-assembled. Herein, we describe four supramolecular self-assembled chair type tetranuclear ruthenium (II) metallacycles containing a flexible diimide based ligand, and their effect on T98G glioblastoma, A549 lung adenocarcinoma, and glioma stem like cells (GSCs). Glioblastoma multiforme (GBM) is the most aggressive type of brain tumor, and accounts for 60% of primary brain tumors, [30] and its dismal prognosis glioma is primarily due to relapse after treatment [31]. This relapse is largely due to the existence of cancer stem-like cells (CSCs), a small sub-population of tumor cells with the ability to self-renew, differentiate, and form secondary tumors [32–35]. CSCs are difficult to eradicate by conventional chemotherapy or radiotherapy, [36] and although recent anticancer therapies successfully reduce tumor load by destroying large numbers of cancer cells, intratumoral CSCs tend to persist. To improve these outcomes, treatment strategies with the potential to kill tumor cells and CSCs are needed. Growing evidence suggests targeting cell-surface markers [37] and signaling pathways [38] offer means of killing CSCs cell death, but as yet no clinically approved drug that specifically targets CSCs has been developed. Accordingly, there is an urgent need for compounds that selectively target cancer cells and their CSCs.

## RESULTS AND DISCUSSION

### Construction and characterization of flexible, ligated, supramolecular self-assembled chair type tetranuclear ruthenium (II) metallacycles

Our emphasis was on the construction of supramolecular coordination driven self-assembly systems based on flexible ditopic N,N'-bis(4-pyridylmethyl)-pyromelliticdiimide ligand (1) and an arene-ruthenium(II) acceptor (A1-A4). In particular, we explored the use of flexible ligands, as opposed to rigid ligands, and their effects on structural features, and examined the conformations of self-assemblies. Here, we describe four arene-ruthenium(II) tetranuclear supramolecular self-assemblies that display selectivity towards brain cancer cells and GSCs. The tetranuclear self-assembled metallacycles (2-5) were obtained using binuclear half-sandwich arene-ruthenium(II) acceptors (A1-A4) and the functionalized flexible ditopicpyromelliticdiimide ligand (1).

**Scheme 1:**
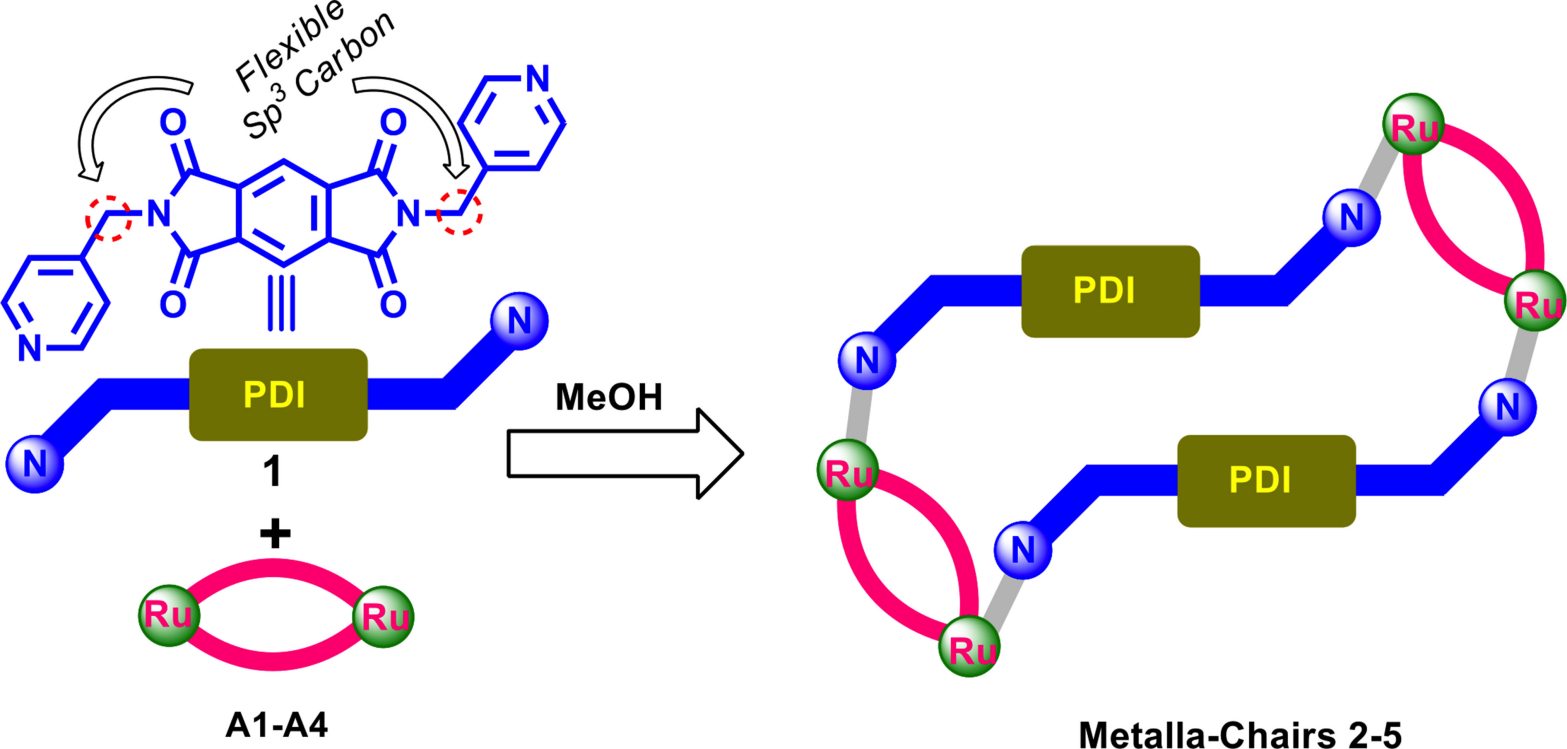
Schematic representation of the synthesis of self-assembled ruthenium(II) assemblies 2-5

**Scheme 2:**
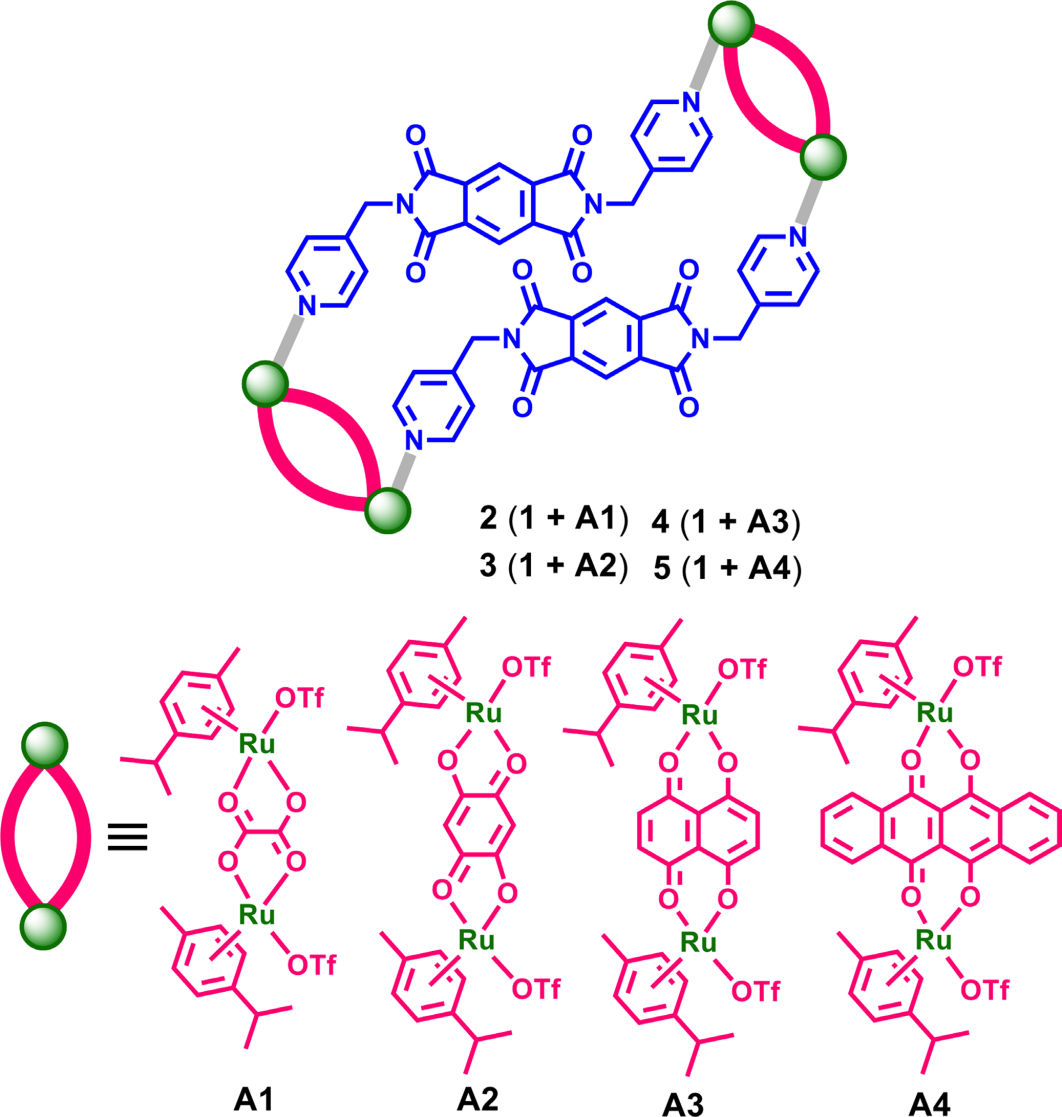
Self-assembled ruthenium (II) metallacycles 2-5

**Figure 1:**
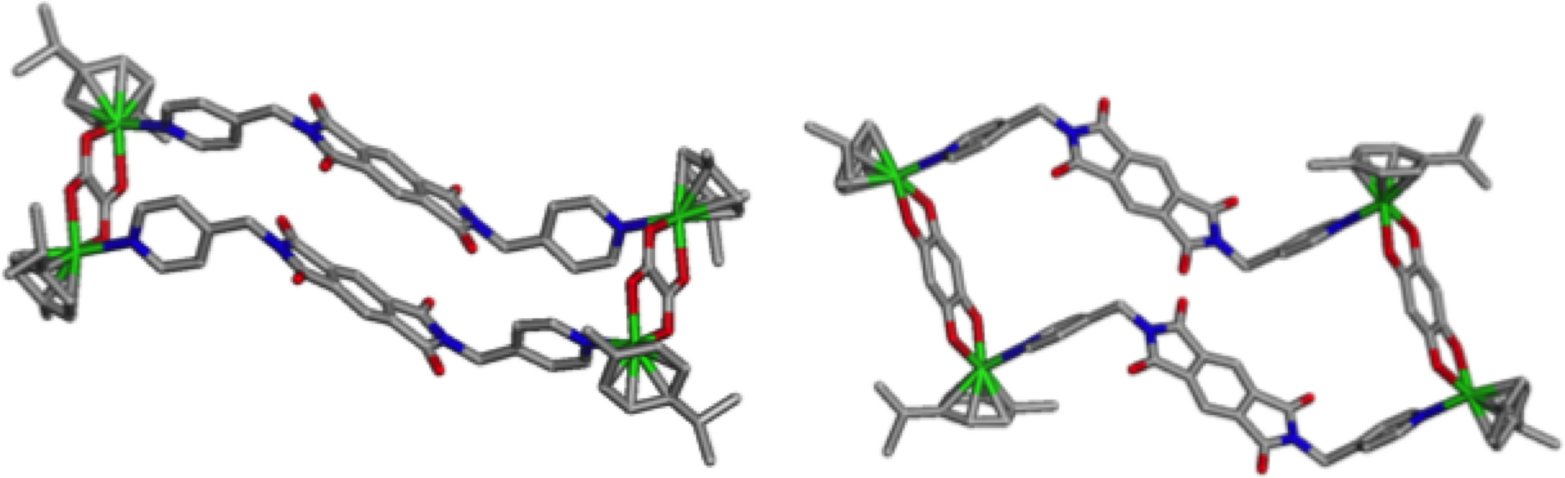
Single Crystal X-ray structures of metallacycles 2 and 3

**Figure 2:**
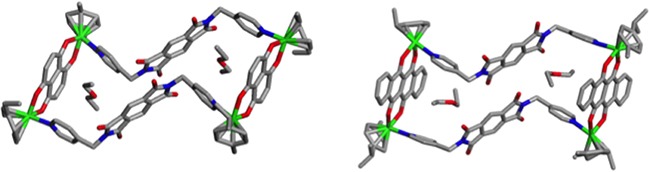
Single Crystal X-ray structures of metallacycles 4 and 5 with encapsulated guest diethyl ether molecules

Supramolecular self-assembled metallacycles 2-5 were synthesized by reacting ditopic flexible pyromellitic diimide N,N'-donor 1 with equimolar amounts of respective arene-ruthenium(II) acceptors A1-A4 in methanol at room temperature for 12 h as shown in Schemes 1 and 2. The metallacycles 2-5 were isolated as pure crystalline materials by adding diethyl ether to concentrated reaction mixtures. These four synthesized metallacycles were fully characterized by NMR spectroscopy (^1^H and ^13^C), high-resolution electrospray mass spectrometry (HR-ESI-MS), and elemental analysis techniques (for detailed results refer to Supporting Information, (SI)). The structural features of the metallacycles were unambiguously confirmed using single crystal X-ray diffraction data of all four metallacycles. Crystals used for SC-XRD analysis were obtained by layering diethyl ether onto methanol solutions of the synthesized metalla self-assemblies at ambient temperature. Single-crystal X-ray diffraction analysis data was obtained using synchrotron radiation at 100 K revealed supramolecular self-assemblies 2-5 adopt tetranuclear chair-shaped structures (Figures 1 and 2).

Coordination spheres around ruthenium metal centers exhibited a slightly distorted octahedral geometry. The dianionic acceptor O,O' units acted as doubly-bridging units as a result of oxygen chelation. Pyromellitic ditopicdiimide ligand pyridine N-donor sites were found to adopt an anti-conformation mode. Two ditopic ligands of 1 were arranged in parallel to form chair-shaped metallacycles. Self-assembled metallacycles were stabilized by various intra- and inter-molecular non-covalent interactions likely π-π stacking interactions between parallel pyromellitic diimide ligand units. In particular, two diethyl ether moieties were capsulated between the two parallel pyridyl moieties of metallacycles 4 and 5, and these moieties were stabilized by other non-covalent interactions such as C−H···O, C−H···F, and C−H···N hydrogen bonds (Figure 2 and Supplementary Materials.).

The ^1^H NMR spectra of the synthesized self-assemblies showed proton resonances were considerably shifted with respect to the starting ligand and the arene-ruthenium(II) acceptors used. In acceptors, alkyl protons were observed as two singlets between 1.39–1.29 ppm for the isopropyl methyl protons and as a singlet in the range 2.22–2.07 ppm for the p-methyl proton. In addition, a multiplet was observed around 2.99–2.75 ppm for the CH-proton of isopropyl moieties. The aryl protons of the p-cymene moieties of metallacycles 2-5 were observed as two doublets at 5.78–5.57, and 6.00–5.81 ppm. The flexible –CH_2_- moieties of ligands were observed at 5.09, 5.73, 4.81, and 4.61 ppm, respectively for 2-5. Full spectroscopic data and a discussion of ^1^H and ^13^C NMR results are provided in Supplementary Figures 1 and 2, Supplementary Materials. Electrospray ionization mass spectroscopy (ESI-MS) was conducted on all four self-assembled products. The presence of prominent multiply charged ions at m/z = 688.0585 [2–3OTf]^3+^, 721.4023, [3–3OTf]^3+^, 754.7463 [4–3OTf]^3+^ and 821.4343 [5–3OTf]^3+^ indicated the formation of [2 + 2] self-assembled products. Observed peaks were isotopically resolved and agreed well with predicted isotopic distribution patterns and matches with reported analogues moieties [14]. ESI-MS spectroscopic data are shown in Supplementary Figure 3, Supplementary Materials.

### Cytotoxicity of self-assembled metallacycles induces actin disruption

**Figure 3:**
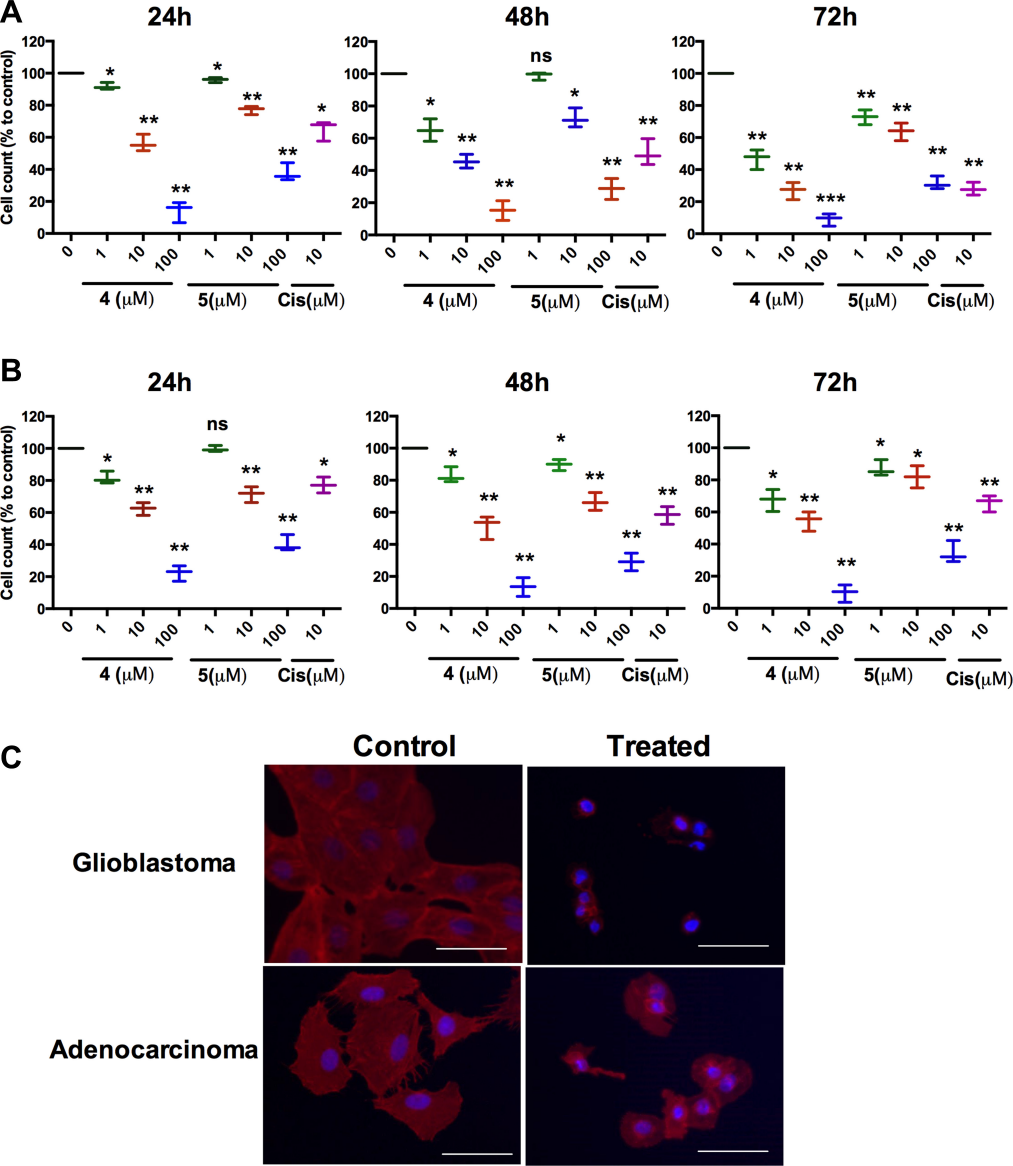
The cytotoxic effects of metallacycles 4 and 5 (**A**) Glioblastoma cell (T98G) viabilities were determined by MTT assay after incubation for 24, 48 and 72 h (h) in DMEM medium. (**B**) Viabilities of lung adenocarcinoma cells (A549) were also determined by MTT assay after 24, 48 and 72 h. (**C**) Immuno fluorescence assays using phalloidinrhodamine (5 units/ml, Invitrogen) were performed to visualize cytoskeletons (F-actin); nuclei were counterstained by DAPI in metallacycle 4 (10 μM) treated glioblastoma and adenocarcinoma cells. Scale bars = 100 μm. Results are expressed as means ± SDs (*n* = 3) of percentages of living cells versus non-treated controls (*p < 0.05 and **p < 0.01 by the Student's *t*-test).

We next investigated whether the synthesized metallacycles had anti-cancer effects. To this end, we tested their effects on human solid tumor cell-lines (T98G glioblastoma and A549 lung adenocarcinoma) and stem-like cancer cells. The viabilities of T98G (glioblastoma) and A549 (lung adenocarcinoma) cell lines were determined using an MTT assay, as previously described, [39–42] inhibitory concentration-50 (IC50) values are listed in Table 1. Of the four self-assembled complexes tested, 4 and 5 potently inhibited tumor cells with IC50 values of 10.5–18.6 μM for T98G and 10.2–39.5 μM for A549 cells (Table 1). Metallacycles 4 and 5 showed concentration-dependent effect in both solid cancer cell lines (Figure 3).

**Table 1:** IC50 values (μM) of starting material and metallacycles

Compound	T98G	A549
**A1**	218.43 (± 34.0)	299.67 (±41.0)
**A2**	163.42 (± 20.5)	182.33 (±19.8)
**A3**	102.5 (± 7.0)	107.27 (±11.3)
**A4**	135.55 (± 28.1)	143.13 (± 4.7)
**1**	225.36 (± 20.3)	308.11 (± 25.4)
**2**	101.52 (± 17.7)	116.83 (± 7.0)
**3**	110.69 (± 9.6)	112.71 (± 11.4)
**4**	10.58 (± 4.7)	10.27 (± 5.2)
**5**	18.64 (± 7.7)	39.58 (± 12.0)
**Cisplatin**	64.7 (± 8.6)	71.2 (± 12.3)

Metallacycles 2-5 and the positive control (cisplatin) as determined by MTT assay on T98G (glioblastoma cells) and A549 (lung adenocarcinoma cells) after 24 h of exposure.

Metallacycle 4 decreased numbers of viable T98G and A549 cells in a time- and dose-dependent manner, whereas metallacycle 5 inhibited cancer growth only in dose-dependent manner (Figure 3A and 3B, respectively). At 24 h both metallacycles were found to be more active than the positive control (cisplatin, 10 μM).

### Stability of self-assemblies 4 and 5

The stabilities of 4 and 5 were examined in human cell culture media over 0–50 h at 37°C. Around 40% reduction in growth inhibitory activity was found for both complexes after incubation for 50 h under physiological conditions, but both 4 and 5 were stable when incubated for < 25 h. These results indicate that 4 and 5 are sufficiently stable under physiological conditions during the early phase of treatment when both have strong effects on human cancer cells (See Supplementary Figure 4 in the Supplementary Materials). To confirm stabilities, 4 and 5 are dissolved in DMSO-d6/D2O (1:1) and subjected to ^1^H NMR immediately after dissolution and then every day for seven days. Their proton resonances were found to remain unchanged for up to seven days (See Supplementary Figures 5 and 6 in the Supplementary Materials).

**Figure 4:**
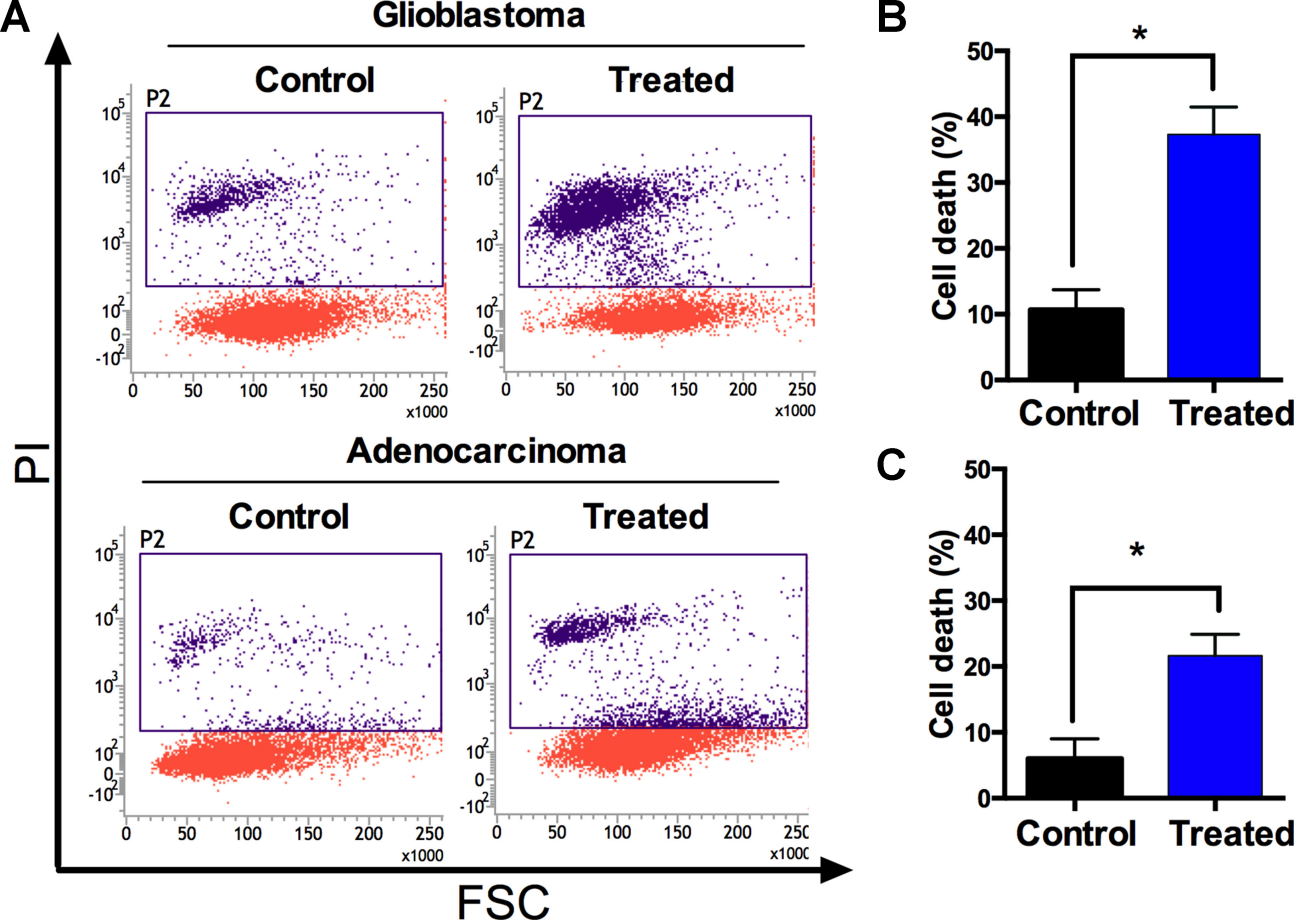
Metallacycle 4 induced cell death in glioblastomaand adenocarcinoma (**A**) Dot plots of cell death analysis findings as determined by propidium iodide (PI) staining/flow cytometry. (**B**) Representative plots of glioblastoma (T98G), and (**C**) of lung adenocarcinoma (A549) cell deaths. Results are expressed as percentages of living cells versus non-treated controls (means ± SDs; *n* = 3).

The actin cytoskeleton plays a critical role in intracellular transport processes and in cancer cell migration, and thus, we immune stained metallacycle 4 treated glioblastoma and lung adenocarcinoma cells for actin to visualize structural changes, as previously described [41, 42]. Interestingly, the dynamics of filamentous actin were altered in both cell lines after treating them with metallacycle 4 (10 μM) for 24 h. Actin fibers were shortened and shrunken after treatment with 4 in both cell lines (Figure 3C), indicating that 4 effectively damaged the cytoskeleton. When cell death was analyzed by propidium iodide (PI) flow cytometry, 30-40% of glioblastoma and 20–30% of adenocarcinoma cells were found to have succumbed (Figure 4A–4C).

### Targeting of glioma stem like cells with metallacycle 4

**Figure 5:**
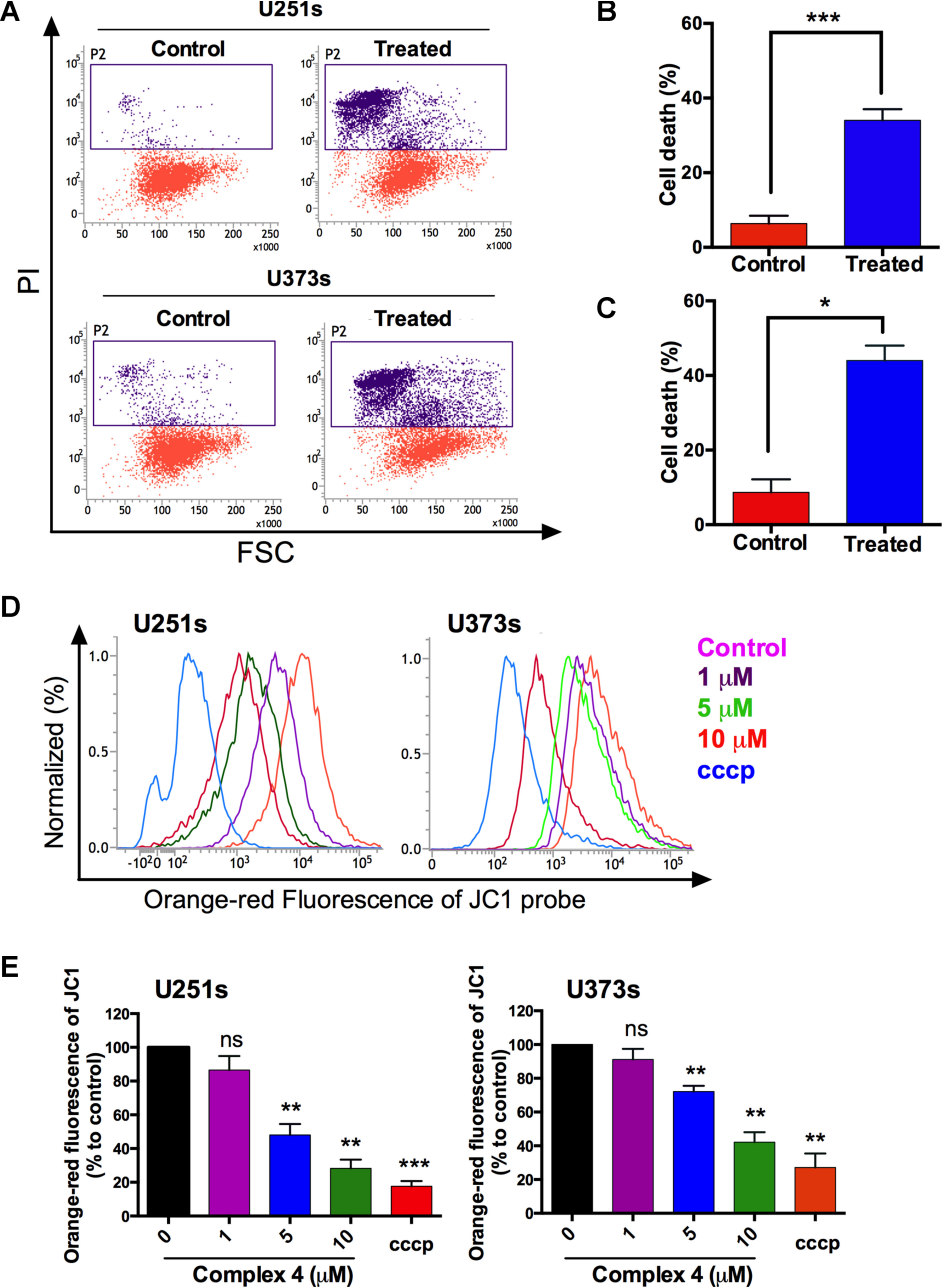
Metallacycle 4 had a cytotoxic effect on glioma stem like cells (**A**) Dot plot of cell death among glioma stem like cells (U251s & U373s gliospheres) as determined by propidium iodide (PI) staining. (**B** and **C**) Representative graph of U251 and U373 cell death. (**D**) Mitochondrial membrane potentials (MMP) were estimated by flow cytometric analyses using JC-1 dye. Orange-red fluorescence intensity curve of cells represent mitocondrial potential, which decreased dose-dependently after treatment with metallacycle 4 at concentrations 1–10 μM. The dark purple curve represents the non-treated control and the blue curve represents the positive control (cccp). The shift from the non-treated control curve to the cccp-treated curve indicates a reduction in MMP. (**E**) Fluorescence relative to the negative control. Results are presented as means ± SDs of three independent experiments, and the analysis was performed using the Student's *t*-test (*p < 0.05 vs. the negative control).

CSCs play crucial roles during the initiation of cancer cell progression, [42] and thus, we examined the effects of the of metallacycle 4 on these cells. Propidium Iodide (PI) staining and flow cytometry showed metallacycle 4 (10 μM for 24 h) induced 35–45% cell death in U251s and U373s glioma-like stems cells (Figure 5A–5C). In addition, mitochondrial membrane potentials (MMP) were examined using JC-1 dye (a cationic dye that accumulates in polarized mitochondria and produces orange-red fluorescence). As a positive control we used the proton ionophore carbonyl cyanide 3-chlorophenylhydrazone (cccp). Figure 5D shows the band shift of JC-1 observed after incubating U251s and U373s cells for 24 h with metallacycle 4. To determine relative changes in mitochondrial membrane potential, they were expressed as percentages of the fluorescence intensities differences observed between those of negative controls (100%) and cccp-treated positive controls (percentage fluorescence intensity changes are plotted in Figure 5E).

**Figure 6:**
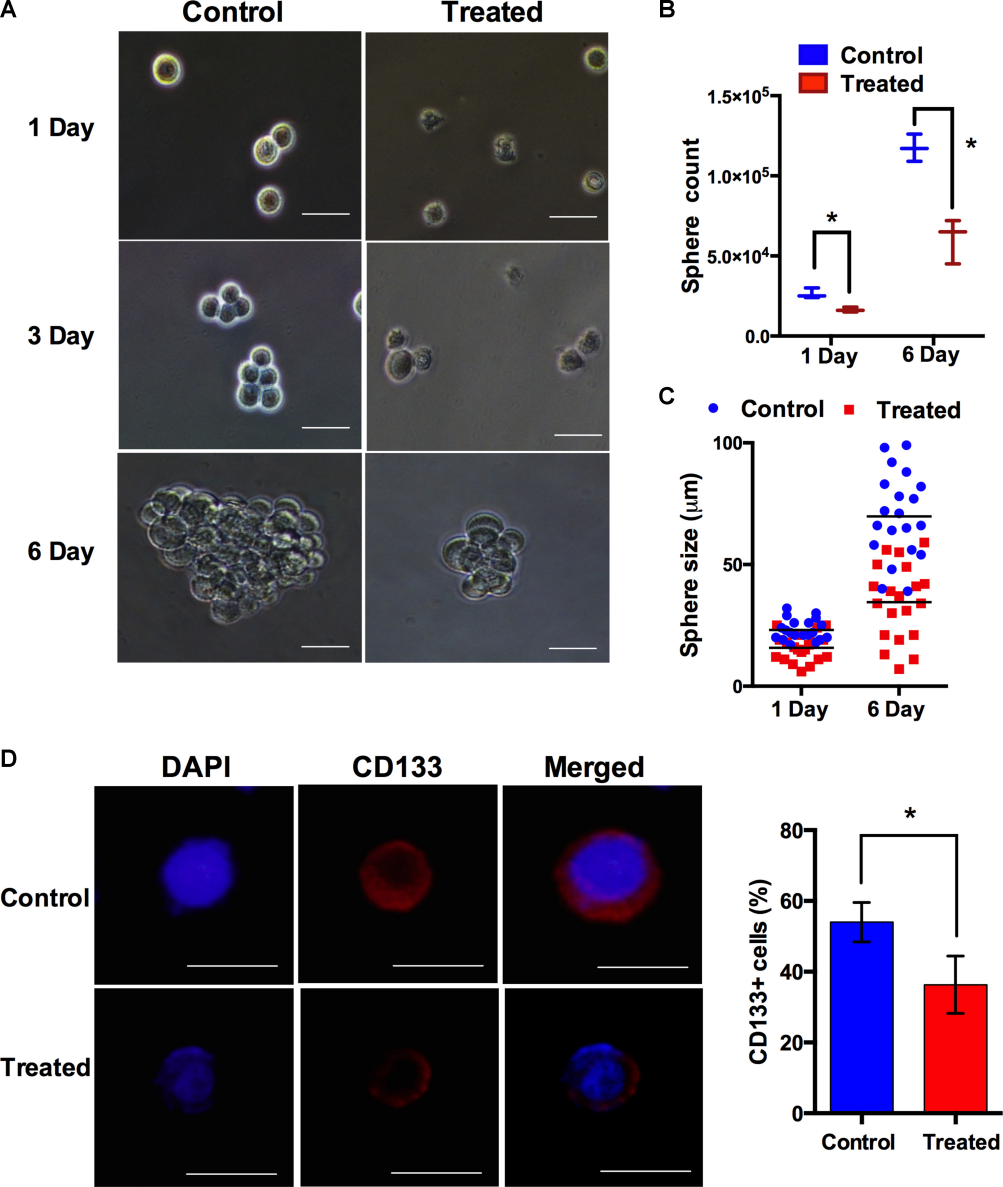
Metallacycle 4 abrogated the sphere-forming ability of glioma stem like cells (U251s) (**A**) Photographs of gliospheres formed by U251s cells after 6 days of metallacycle 4 treatments at 5 μM. (**B**) Quantification of sphere forming ability of U251s cells. (**C**) Graphical presentation of the average sizes of U251 gliospheres, formed after treatment with metallacycle 4 at 5 μM. (**D**) Immunocytochemical analysis for CD133 (a CSC marker in glioma) in U251 spheres grown in the presence/absence of metallacycle 4. Nuclei were counterstained with DAPI (4,6-diamidino-2-phenylindole). CD133 positive cells were quantified by flow cytometry.

Fluorescence results indicated metallacycle 4 (10 μM) significantly attenuated (> 40%) MMPs in U251s and U373s cell lines. We also performed sphere clone formation and self-renewal capacity assays, as described previously [42]. An individual single sphere cell was used to analyze the effect on these CSC characteristics. Sphere formation analysis revealed that sphere numbers were significantly reduced after 6 days of treatment with metallacycle 4 (5 μM) as shown in Figure 6A and 6B.

We also observed that treatment with metallacycle 4 (5 μM) reduced the ability of gliospheres to grow (Figure 6C). Consistent with this observation, we found the enrichment of CD133+ cells in U251s gliospheres, as compared with monolayer culture, indicating the presence of CSCs. Interestingly, treatment with metallacycle 4 significantly decreased the number of CD133+ cells in U251s sphere-cultured cells. Immunocytochemical analysis also demonstrated that metallacycle 4 treatment decreased the expression of CD133 in gliospheres (Figure 6D). Taken together, these results indicate metallacycle 4 efficiently suppresses malignant phenotype of glioma stem like cells, and that self-assembled synthesized metallacycles are able to suppress cancer progression and inhibits the growth of cancer-like stem cells.

Ruthenium based complexes promise to provide alternatives to platinum-based drugs for anticancer therapy [43, 44]. Ruthenium complexes as anticancer drugs are almost always designed to mimic platinum drugs, for targeting DNA [45]. Action of mechanism of ruthenium complexes for anticancer activity is mostly based on (i) activation by reduction [43] (ii) Via transferrin or albumin interactions [45]. Transferrin and albumin are good binding proteins to Ruthenium complexes and stronger affinity for cancer tissues over normal tissues [46]. The Ru (II) complexes also capable to form adducts with guanine and have a higher binding affinity towards DNA in the presence of protein than the Ru(III) compounds [47]. Recent study also shows ruthenium complexes can block cell cycle progression and induces reactive oxygen species-reliant and mitochondria-based apoptosis [48]. It can also activate Nrf2 pathway for apoptosis primarily through the activation of caspase [49]. Another important report suggest that Ru-arene complexes are promising anti-cancer drugs that inhibit progression and metastasis by blocking multiple processes of cancers [50]. These Ru-arene complexes also inhibited growth factor productions such as VEGF-A, PDGF-AA, and GM-CSF proteins at the transcriptional levels. These all reported mechanism events such as reactive species production, mitochondria stress, DNA damage, interaction with transferrin and albumins, and inhibition of various kinds of growth factors, are also responsible for the CSCs (CD133 or CD44 specific cells) selectivity by ruthenium complexes.

## MATERIALS AND METHODS

### General Details

The chloride derivative of arene-ruthenium acceptor A1-A4 [51] and corresponding triflate derivative [52], N,N'-bis(4-pyridylmethyl)-pyromellitic diimide ligand 1 [53] and ruthenium (II) metallacycles 2-4 were prepared according to reported and/or modified methods [14]. Deuterated NMR solvents were purchased from Cambridge Isotope Laboratory (Andover, MA, USA). NMR spectra were recorded on Bruker 300 M Hz. ^1^H NMR chemical shifts are reported relative to the residual protons of deuterated CD_3_OD (3.31 ppm) and deuterated CD_3_NO_2_ (4.33 ppm). ESI-MS data were recorded on Synapt G2 quadrupole time-of flight (TOF) mass spectrometer equipped with an electrospray ion source (Waters, Milford, MA, USA) and analyzed with the MassLynx software suite system at the Korea Basic Science Institute, Ochang).

### Single crystal X-ray diffraction

Intensity data of suitably sized crystals of self-assemblies 2-5 (CCDC: 1498700-1498703) were collected at 100 K on an ADSC Quantum 210 CCD diffractometer with synchrotron radiation (λ = 0.70000 Å) at the Supramolecular Crystallography Beamline 2D, Pohang Accelerator Laboratory (PAL), Pohang, Korea. The raw data were processed and scaled using the program HKL2000. The structure was solved by direct methods, and the refinements were carried out with full-matrix least-squares on F^2^ with appropriate software implemented in the SHELXTL program package [54]. All the non-hydrogen atoms were refined anisotropically, and hydrogen atoms were added to their geometrically ideal positions. The contributions of the most disordered solvent molecules were removed from the diffraction data using the SQUEEZE routine of PLATON software, [55, 56] and then final refinements were carried out.

### Synthesis of metallacycle 2

The solution of Ru acceptor A1 (8.57 mg, 0.01 mmol) in methanol (1.0 ml) was added drop wise to the solution of donor ligand 1 (3.98 mg, 0.01 mmol) in methanol (1.0 ml). The reaction mixture was stirred overnight at room temperature and filtered to obtain a clear solution which was concentrated to 0.5 ml. To this concentrated solution diethyl ether was added drop wise to obtain metallacycle 2 as yellow colored precipitate which was collected and dried after centrifugation in good yield 84% (10.54 mg). Anal. Calcd for C_92_H_84_F_12_N_8_O_28_Ru_4_S_4_·2H_2_O: C, 43.40; H, 3.48; N, 4.40. Found: C, 43.16; H, 3.38; N, 4.51. ^1^H NMR [300 MHz, CD_3_OD]: δ (ppm) 8.04 (d, J = 6.7 Hz, 8H, H^a^), 7.99 (s, 4H, H^c^), 7.42 (d, J = 6.6 Hz, 8H, H^b^), 5.94 (d, J = 6.3 Hz, 8H, H^4^), 5.77 (d, J = 6.4 Hz, 8H, H^3^), 5.09 (s, 8H, H^d^), 2.82 (m, 4H, H^2^), 2.22 (s, 12H, H^5^), 1.36 (d, J = 6.9 Hz, 24H, H^1^); ^13^C NMR [75 MHz, CD_3_OD]: δ (ppm) 172.08, 167.77, 153.34, 151.38, 138.73, 124.93, 119.87, 103.56, 98.98, 83.89, 83.16, 41.59, 32.63, 22.62, 18.19; ESI-MS for 2: m/z = 688.0585 [M – 3OTf]^3+^.

### Synthesis of metallacycle 3

Metallacycle 3 was synthesized following the same procedure of 2 with ruthenium acceptor A2 (9.07 mg, 0.01 mmol). The complex 3 was obtained as red crystalline solid in 87% (11.35 mg) yield. Anal. Calcd for C_100_H_88_F_12_N_8_O_28_Ru_4_S_4_·2H_2_O: C, 45.39; H, 3.50; N, 4.23. Found: C, 45.10; H, 3.45; N, 4.52. ^1^H NMR [300 MHz, CD_3_OD]: δ (ppm8.43 (s, 4H, H^c^), 8.24 (d, J = 5.6 Hz, 8H, H^a^), 7.47 (d, J = 5.8 Hz, 8H, H^b^), 5.99 (d, J = 6.0 Hz, 8H, H^4^), 5.77 (d, J = 5.9 Hz, 8H, H^3^), 5.73 (s, 8H, H^d^), 4.90 (s, 8H, H^d^), 2.85 (m, 4H, H^2^), 2.16 (s, 12H, H^5^), 1.34 (d, J = 6.9 Hz, 24H, H^1^); ^13^C NMR [75 MHz, CD_3_OD]: δ (ppm) 185.60, 167.60, 154.17, 151.27, 138.85, 126.12, 119.91, 105.36, 102.83, 100.05, 84.87, 83.44, 41.40, 32.72, 22.65, 18.27; ESI-MS for 3: m/z = 721.4023 [M – 3OTf]^3+^.

### Synthesis of metallacycle 4

Metallacycle 4 was synthesized following the same procedure of 2 with ruthenium acceptor A3 (9.57 mg, 0.01 mmol). The complex 4 was obtained as old copper crystalline solid in 94% (12.74 mg) yield. Anal. Calcd for C_108_H_92_F_12_N_8_O_28_Ru_4_S_4_·2H_2_O: C, 47.23; H, 3.52; N, 4.08. Found: C, 47.13; H, 3.36; N, 4.23. ^1^H NMR [300 MHz, CD_3_OD]: δ (ppm) 8.42 (s, 4H, H^c^), 8.37 (d, J = 6.5 Hz, 8H, H^a^), 7.39 (d, J = 6.5 Hz, 8H, H^b^), 7.17 (s, 8H, H^6^), 5.80 (d, J = 6.3 Hz, 8H, H^4^), 5.58 (d, J = 6.3 Hz, 8H, H^3^), 4.81 (s, 8H, H^d^), 2.79 (m, 4H, H^2^), 2.07 (s, 12H, H^5^), 1.30 (d, J = 6.9 Hz, 24H, H^1^); ^13^C NMR [75 MHz, CD_3_OD]: δ (ppm) 172.40, 167.58, 153.29, 150.87, 138.82, 138.66, 126.06, 119.81, 112.72, 105.06, 101.03, 85.83, 84.11, 41.33, 32.12, 22.59, 17.48; ESI-MS for 4: m/z = 754.7463 [M – 3OTf]^3+^.

### Synthesis of metallacycle 5

Metallacycle 5 was synthesized following the same procedure of 2 with ruthenium acceptor A4 (9.07 mg, 0.01 mmol). The complex 5 was obtained as red crystalline solid in 93% (13.53 mg) yield. Anal. Calcd for C_124_H_100_F_12_N_8_O_28_Ru_4_S_4_: C, 51.17; H, 3.46; N, 3.85. Found: C, 50.92; H, 3.45; N, 3.96. ^1^H NMR [300 MHz, CD_3_OD]: δ (ppm) 8.65 (dd, J = 6.1, 3.3 Hz, 8H, H^6^), 8.51 (d, J = 6.6 Hz, 8H, H^a^), 8.17 (s, 4H, H^c^), 7.88 (dd, J = 6.5, 3.3 Hz, 8H, H^7^), 7.22 (d, J = 6.7 Hz, 8H, H^b^), 5.89 (d, J = 6.3 Hz, 8H, H^4^), 5.69 (d, J = 6.3 Hz, 8H, H^3^), 4.61 (s, 8H, H^d^), 2.94 (m, 4H, H^2^), 2.20 (s, 12H, H^5^), 1.32 (d, J = 6.9 Hz, 24H, H^1^); ^13^C NMR [75 MHz, CD_3_OD]: δ (ppm) 170.56, 167.34, 153.07, 149.78, 138.33, 134.86, 134.02, 128.11, 126.06, 119.45, 108.22, 105.07, 100.54, 84.88, 83.75, 40.98, 31.83, 22.42, 17.76; ESI-MS for 5: m/z = 821.4343 [M – 3OTf]^3+^.

### Human cell culture maintenance and treatment

Human cancer cell lines T98G (glioblastoma), A549 (lung adenocarcinoma) were obtained from Korean Cell Line Bank and cultured in DMEM media (Welgene, Korea) supplemented with 10% fetal bovine serum and 1% penicillin/streptomycin solution (Sigma-Aldrich). The U-251 MG and U-373 MG cell lines were also obtained from Korean Cell Line Bank (Seoul, Korea) and cultured in DMEM. To prepare glioma like stem cells in condition media containing DMEM-F12 media with FGF, EGF and B27 supplement (Gibco, Korea) from culture of U251 and U373 cell lines. The prepared gliosphere (glioma like stem cells) cultures were maintained in condition media for further experiments. All cultures were maintained at 37°C, 95% relative humidity and 5% CO_2_. Cells were seeded in 12-well plates at a concentration of 1 × 10^5^ cells/well in 1mL of complete media and incubated for 24 h (hr) at 37°C in 5% CO_2_ atmosphere to allow for cell adhesion. Stock solutions (5 mg/mL) of complexes and starting materials diluted in PBS and were filter-sterilized, and a specific volume of sub-stock solution is added to a fresh medium in wells to produce various final concentrations. Control group containing no complexes or compounds was run in triplicates during each assay.

### Quantification of viability and cell death

Viability was measured by well-known MTT assay. Cell death was determined by BD FACSVerse flow cytometer, using FACS suite software. Briefly, cells were harvested by trypsinization, washed in PBS, and then incubated in propidium iodide at concentration of 10 μg/mL for 5 min at room temperature and 10,000 events/sample were analyzed [39–41, 57]

### Assessment of the stability of the complexes

For evaluation of the stability of 4 and 5 synthesized complexes, stability was checked from 0 to 50 h in physiological media solutions. Aqueous solutions with DMEM media were used to test the stability of the complexes. The stability of complexes was evaluated by using the MTT assay to check metabolic growth inhibition in glioblastoma tumor cells (See Supplementary Figure 4).

### Immunofluorescence

Actin fibers and the nucleus were stained after 24 h of treatments. Cells grown on cover glass were washed with DPBS (Welgene, Korea), fixed with 4% paraformaldehyde (Gentic, USA) for 20 min, and permeabilized by 0.1% of Triton X-100 (Sigma-Aldrich, Korea) in PBS for 25 min at room temperature. After washing with DPBS, actin fibers were stained by phalloidin dye (5 units/ml, Phallotoxins; Invitrogen) for 30 min at room temperature. After washing thrice with DPBS, the samples were mounted with the Prolong gold anti-fade reagent with DAPI (Molecular probes; Invitrogen) and observed under fluorescence microscope (Ti-U, Nikon).^42^ For CD133 expression, after dissociation with accutase, gliospheres cells were stained with anti-CD133/1-PE (AC133 clone; Miltenyi Biotec, USA)^42^ and further processed as described above.

### Flow cytometric analysis of mitochondrial membrane potential

To detect changes in the mitochondrial membrane potential after treatments, JC-1 staining was performed on treated cells with MitoProbe JC-1 assay kit (Invitrogen, Carlsbad, CA, USA). JC-1 is a sensitive marker for the detection of the mitochondrial membrane potential in apoptotic cells. JC-1 shows green fluorescence (due to the depolarized mitochondrial membrane) inside the cell. However, normal polarized mitochondrial membrane cells show red fluorescence. For positive MMP depolarization, we used cccp, which abolishes the membrane potential quickly. The protophore cccp mainly induces mitochondrial permeability transition in cancer cells. In our experiment, we compare our results with cccp as control. Therefore it may be possible that metalla cycles can induce transient nature of mitochondrial membrane potential change in cancer cells [58].

### Sphere colony formation, self-renewal assays and CD133+ cell detection

Briefly, post treatment of sphere-cultured glioma cells with metallacycle, sphere size was monitored at day 1 and 6 by using Motic Images Plus 2.0 in three randomly chosen fields. For clonal assay, glioma sphere cells were plated into 96-well plates at a density of 1 individual cell each well. After 1 day, each well was visually checked for the presence of a single cell. The clones were grownup and clone formation was monitored at 1 and 6 days. Numbers of clones were counted under microscope [42]. For self-renewal assay, individual cell was seeded in each well of 96 well plate and the size of sphere was measured under phase-contrast microscope [42]. For CD133 expression, after dissociation with accutase, gliospheres cells (5 × 10^5^) were labeled with anti-CD133/1-PE (AC133 clone; Miltenyi Biotec, Auburn, CA, USA). For each sample the respective control was prepared without antibody staining. All samples were incubated for 20 minutes at 4°C, washed twice with PBS and immediately analyzed using a BD FACSVerse cytometer and the FACS suite software [42].

### Statistical analysis

All data were expressed as the mean ± SD of triplicates. Significant differences between groups were analyzed by Student's *t*-test. The results were considered statistically significant at *p* < 0.05.

## CONCLUSIONS

We describe a series of self-assembled, flexible, arene-ruthenium (II) based, cationic tetranuclear coordination-driven chair-type metallacycles. These tetranuclear self-assemblies were synthesized and characterized using spectroscopic and analytical techniques. In addition, single crystal X-ray diffraction was used to determine structures and the mode of ligand coordination. The self-assembled metallacycles 2-5 were screened for their *in vitro* anticancer activities and among 4 and 5 were found to be highly effective at killing glioma and adenocarcinoma cells. Metallacycle 4 was screened for its anti-CSC effect against GSCs containing high CD133-positive population. GSCs death was confirmed by PI/flow cytometry and this observation was supported by metallacycle 4 induced reductions in MMPs. Metallacycle 4 also inhibited the sphere forming and self-renewing abilities of GSCs. Furthermore, immune cytochemistry and flow cytometric analysis for CD133 (a CSCs surface marker in glioma) showed that metallacycle 4 altered GSC phenotype. Overall our data suggest metallacycle 4 has the potential to eliminate CSCs in solid cancers and that it has effective anti-CSC activity in GSCs. A detailed preclinical study has been initiated to determine the mode of action of metallacycle 4 and to investigate safety issues.

## SUPPLEMENTARY MATERIALS FIGURES



## Abbreviations

GSCs: Glioma stem like cells
GBM: Glioblastoma multiforme
CSCs: cancer stem-like cells
HR-ESI-MS: High-resolution electrospray mass spectrometry
ESI-MS: Electrospray ionization mass spectroscopy
MMP: Mitochondrial membrane potentials
cccp: Carbonyl cyanide 3-chlorophenylhydrazone (cccp)
IC50: inhibitory concentration-50
PI: Propidium Iodide
TOF: time-of flight
PAL: Pohang Accelerator Laboratory
