# Supplementary material for: Flexible ligated ruthenium(II) self-assemblies sensitizes glioma tumor initiating cells in vitro
Source: Oncotarget. 2017 Jul 5;8(36):60188–200. doi: 10.18632/oncotarget.19028 (PMC5601131; doi:10.18632/oncotarget.19028)
Supplement: Supplementary file 1 [file oncotarget-08-60188-s001.pdf]

Flexible ligated ruthenium(II) self-assemblies sensitizes glioma tumor initiating cells *in vitro*

SUPPLEMENTARY MATERIALS

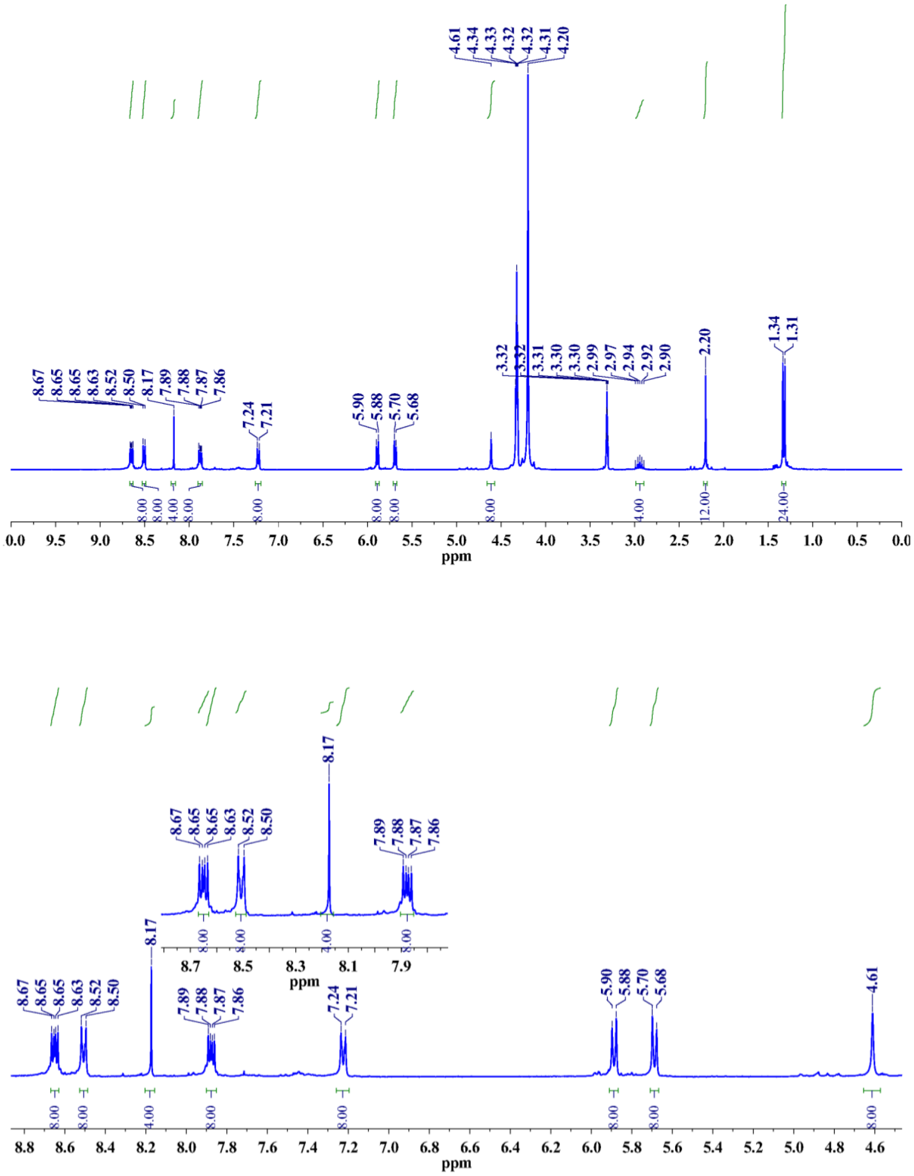

Supplementary Figure 1: The ^{1}H NMR spectra of metallacycle 5 in CD3OD and CD_3NO_2.

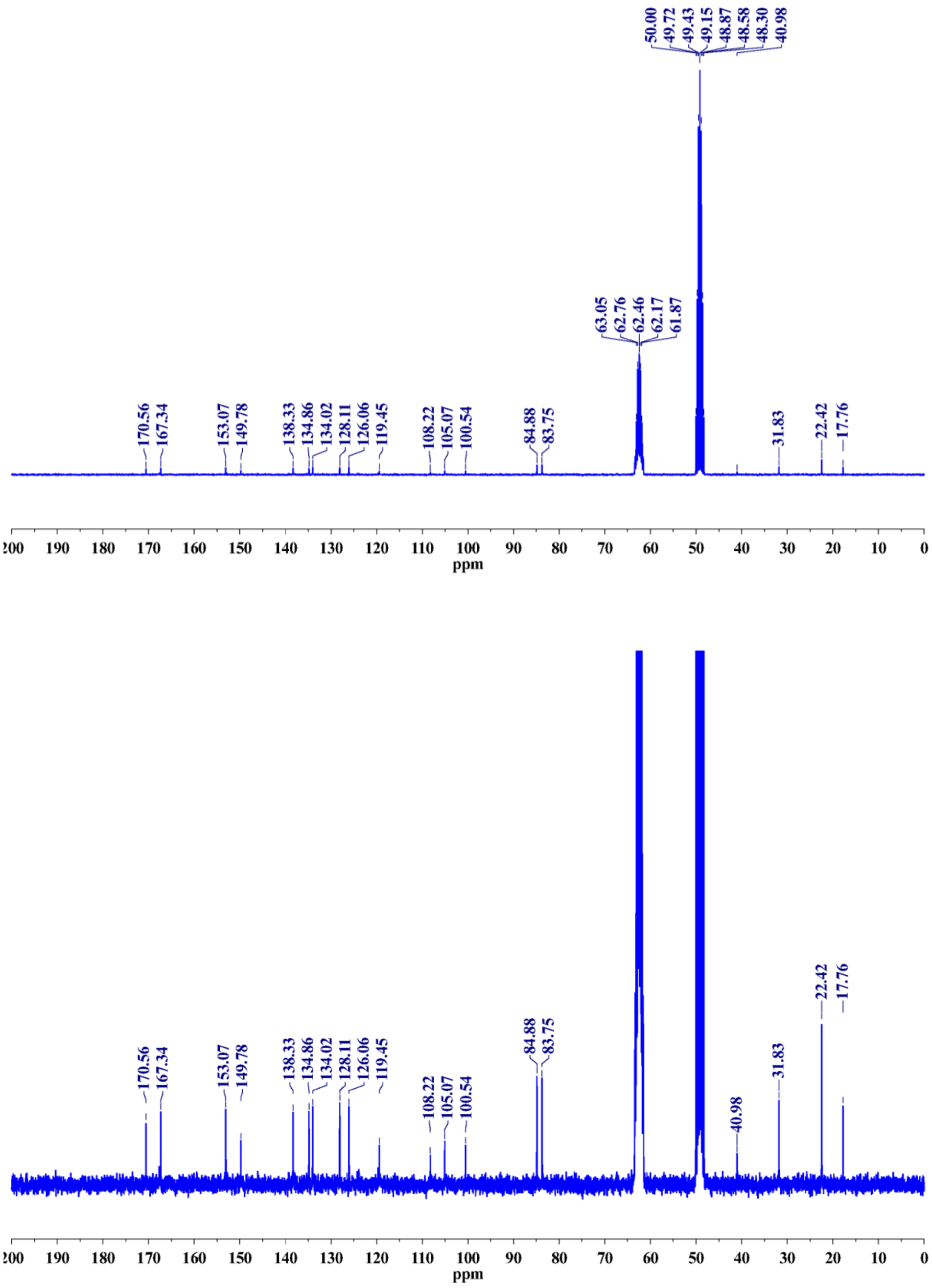

Supplementary Figure 2: The ^{13}C NMR spectra of metallacycle 5 in CD_3OD.

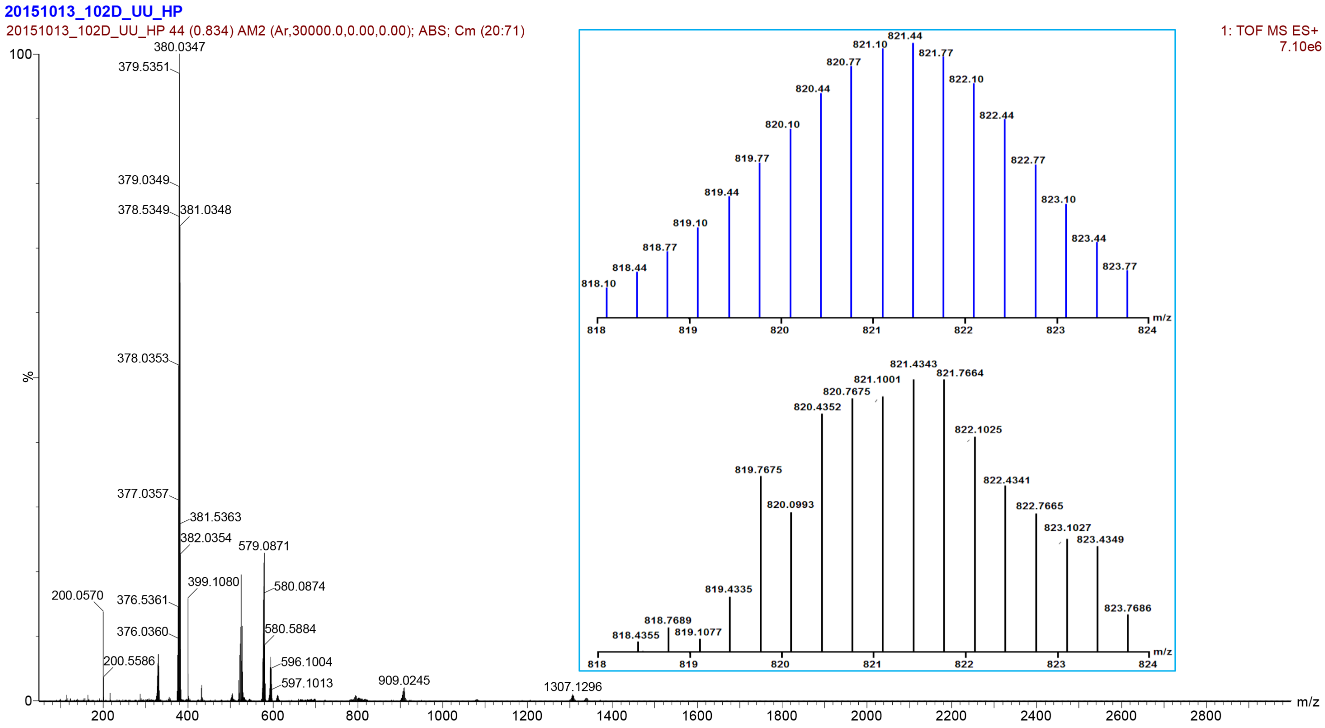

Supplementary Figure 3: The ESI-MS spectrum of metallacycle 5; *insert*: Calculated (blue) and experimental (black) for metallacycle 5 [M –3OTf]$^{3+}$.

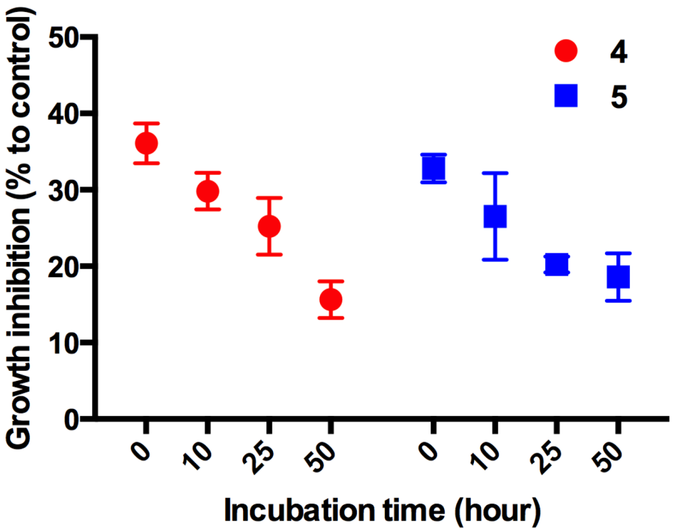

Supplementary Figure 4: Loss of growth inhibitory activity of the complexes pre-incubated in culture media. Self-assemblies 4 and 5, at concentration of 5 µm, were pre-incubated in dmem cell culture medium supplemented wit 10% fbs at 37°C for the indicated times before being added to cultures of glioblastoma cells.

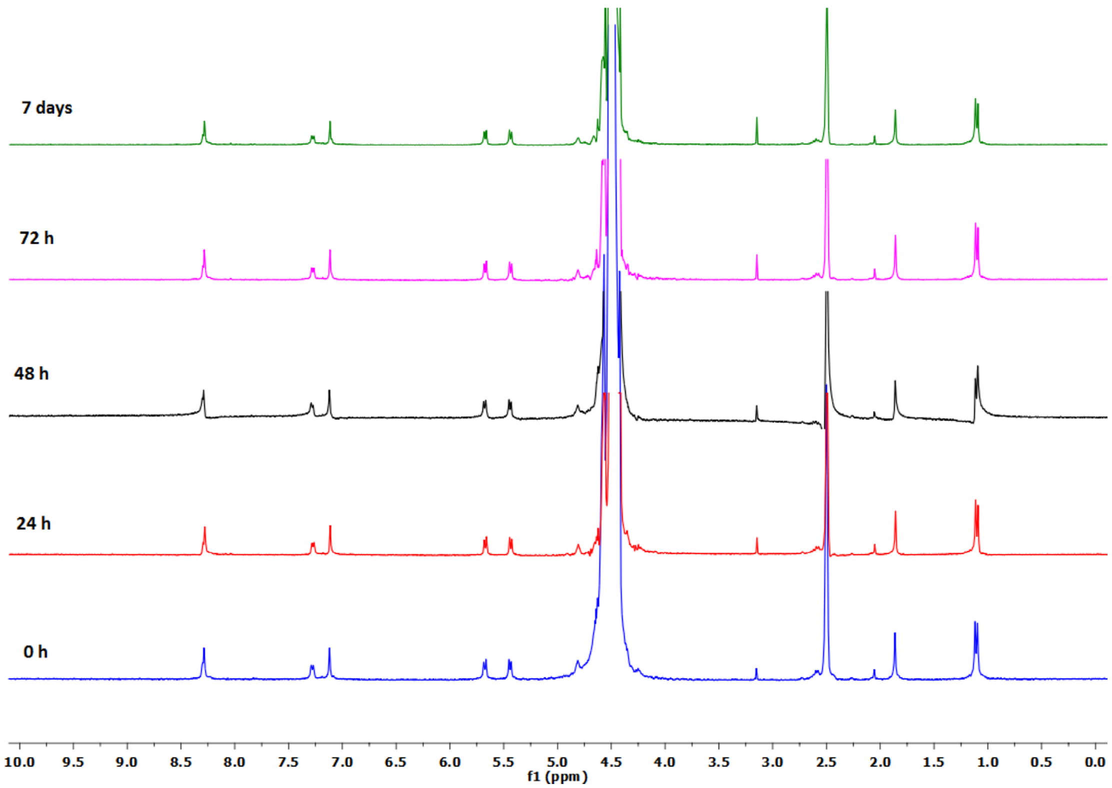

Supplementary Figure 5: The ^{1}H NMR spectra of metallacycle 4 for 0, 24, 48 h and 7 days in DMSO-d_6/D_2O (1:1).

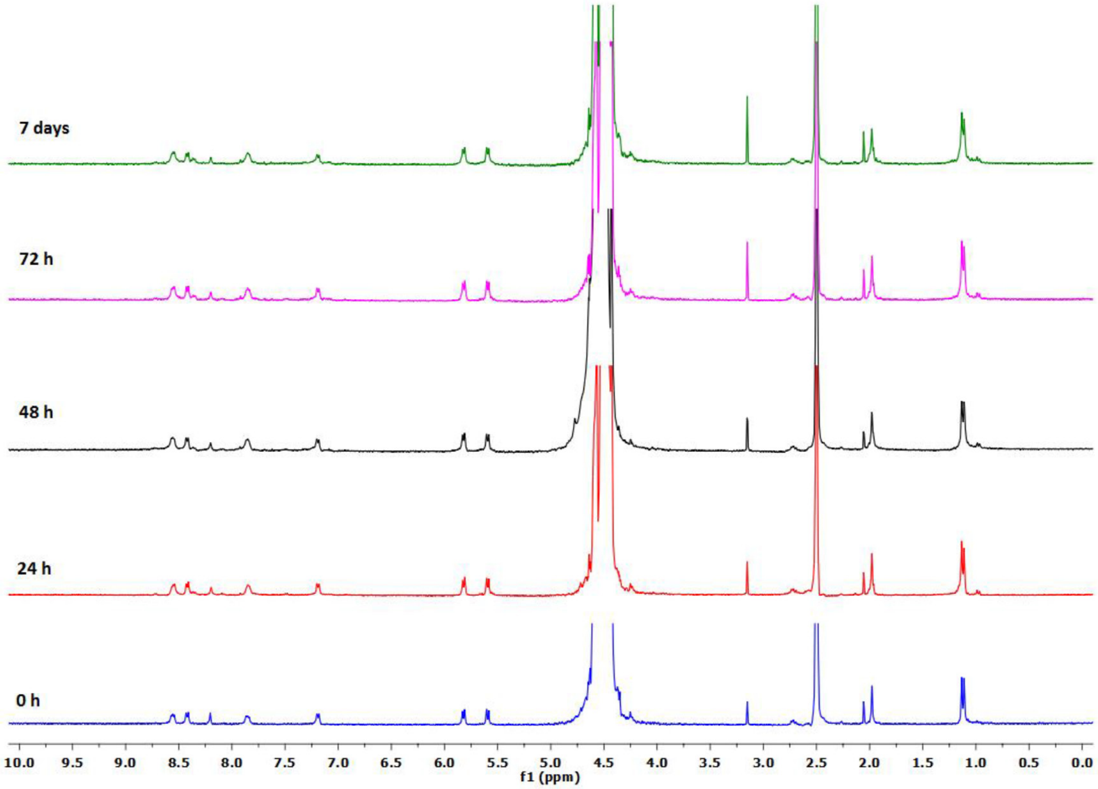

Supplementary Figure 6: The ^{1}H NMR spectra of metallacycle 5 for 0, 24, 48 h and 7 days in DMSO-d_6/D_2O (1:1).
